# Dapagliflozin Versus Vildagliptin as an Adjuvant to Metformin in Patients With Type 2 Diabetes Mellitus: A Randomized, Open-label Study

**DOI:** 10.7759/cureus.38200

**Published:** 2023-04-27

**Authors:** Kumar Gautam, Ratikanta Tripathy, Dayanidhi Meher, Jyoti Prakash Sahoo

**Affiliations:** 1 Pharmacology, Kalinga Institute of Medical Sciences, Bhubaneswar, IND; 2 Endocrinology, Kalinga Institute of Medical Sciences, Bhubaneswar, IND

**Keywords:** euglycemia, antidiabetic, glucose metabolism, glycated haemoglobin, hyperglycemia., metformin therapy, sodium-glucose cotransporter-2, dipeptidyl peptidase-4

## Abstract

Introduction: The rising burden of diabetes mellitus led to the development of novel drugs like dapagliflozin and vildagliptin. Their efficacies in chronic diabetic patients have been thoroughly studied. However, there is a paucity of comparative studies on these drugs in newly diagnosed diabetic patients. The endpoints of our study were changes in glycated hemoglobin (HbA_1c_), fasting blood glucose (FBG), and postprandial blood glucose (PPBG) at 24 weeks from baseline.

Methods: This randomized, open-label, 24-week study was conducted at Kalinga Institute of Medical Sciences, Bhubaneswar, India, from January 2021 to November 2022. The participants were randomized in a 1:1 ratio to receive tablets of either dapagliflozin 10mg once daily or vildagliptin 50mg once daily as an add-on to metformin 500-2000 mg. The analyses were performed in the per-protocol population. We used R software v. 4.1.1 (R Foundation, Indianapolis, IN) for data analysis.

Results: 114 (83.8%) of 136 enrolled participants completed this study. The mean age of the study population was 41.08±5.17 years. Additionally, 52 (45.6%) of them were females. The mean changes in HbA_1c_ from baseline were -1.19 (95% CI: -1.36 to -1.03) and -1.28 (95% CI: -1.37 to -1.18) in dapagliflozin and vildagliptin groups, respectively (p=0.21). The median changes in FBG and PPBG in both groups were -38.76, -46.13 (p=0.07), and -51.84, -53.56 (p=0.14), respectively.

Conclusions: Reductions in HbA_1c_, FBG, and PPBG with add-on treatment of vildagliptin were more pronounced than dapagliflozin after a 24-week intervention. However, the differences were not statistically significant.

## Introduction

The global incidence of type 2 diabetes mellitus (T2DM) has skyrocketed in the last 20 years [1,2]. It has affected large swathes of the population worldwide regardless of age, race, ethnicity, and socioeconomic status. India ranks second in the global epidemiology of diabetes [3]. T2DM accounts for over 90% of all diabetic cases in India [2]. According to an estimate, over 74 million Indians were diagnosed with diabetes in 2021, and this number is expected to rise to over 124 million by 2045 [4]. Despite several antidiabetic medications, sustained glycemic control, complications, and compliance remain unresolved for decades. Hence, there remains a constant requirement for novel drugs to tackle these issues [5].

Metformin is prescribed ubiquitously by endocrinologists to tackle type II diabetes mellitus unless contraindicated. It inhibits the mitochondrial respiratory chain in the liver, leading to activation of AMP-activated protein kinase (AMPK), enhancement of insulin sensitivity (via effects on fat metabolism), and lowered cyclic adenosine monophosphate (cAMP) levels, thereby reducing the expression of gluconeogenic enzymes and maintaining euglycemic state [6]. By inhibiting sodium-glucose co-transporter 2 (SGLT2), dapagliflozin blocks the reabsorption of filtered glucose in the kidney, increasing urinary glucose excretion and reducing blood glucose levels. Its mechanism of action is independent of pancreatic β cell function and modulation of insulin sensitivity [7]. Vildagliptin binds covalently to the catalytic site of dipeptidyl peptidase-4 (DPP-4), eliciting prolonged enzyme inhibition. This raises intact glucagon-like peptide-1 (GLP-1) levels, both after meal ingestion and in the fasting state. It has been shown to stimulate insulin secretion and inhibit glucagon secretion in a glucose-dependent manner [8]. Recent studies [9,10] evaluated the efficacy of these drugs as an adjuvant to metformin monotherapy in managing T2DM and provided inconsonant results. Lately, a network meta-analysis [11] showcased the improved efficacy of add-on vildagliptin and dapagliflozin to metformin monotherapy as compared to antidiabetic drugs in the long-term management of diabetes.

Since none of the previous studies have provided an irrefutable verdict regarding any of these two drugs in newly diagnosed diabetic patients, we planned this study to determine the efficacy of dapagliflozin versus vildagliptin as an add-on to metformin for 24 weeks in the patients with newly diagnosed T2DM.

## Materials and methods

This prospective, interventional, two-arm, parallel-group, randomized, open-label, 24-week study was conducted from 3rd January 2021 to 20th November 2022, involving patients with T2DM in the departments of Pharmacology and Endocrinology, Kalinga Institute of Medical Sciences, Bhubaneswar, India. We obtained approval (KIIT/KIMS/IEC/495/2020, dated 03.11.2020) from the Institutional Ethics Committee, Kalinga Institute of Medical Sciences, Bhubaneswar, before the enrolment of the first participant. It was conducted following the Declaration of Helsinki and the principles of Good Clinical Practice. The patient information sheet narrating the purpose of the study and the risk-benefit details for the participants was provided in the local vernacular language to each patient screened for eligibility. Written informed consent was obtained from all the study participants prior to their enrolment. This study was prospectively registered with the Clinical Trial Registry, India (CTRI/2020/12/030147).

Newly diagnosed T2DM patients with baseline HbA1c ≥ 7.5 and willing to provide written informed consent for participation were included in this study. The persons diagnosed with type 1 diabetic mellitus, pre-existing congestive heart failure, chronic kidney disease, pancreatitis, any malignant condition, those with a history of thromboembolic stroke, myocardial infarction, deep vein thrombosis in the last six months, those with estimated glomerular filtration rate (eGFR) ≤ 45 ml/min/m2, those with elevated liver enzymes or serum bilirubin levels, pregnant or lactating women were excluded from the study.

We enrolled the eligible patients and randomly assigned them via a computer-generated randomization sequence in a 1: 1 ratio to receive either dapagliflozin or vildagliptin as an adjuvant to metformin for 24 weeks. We stratified the randomization based on the gender (male or female) and age (≤ 50 or > 50 years) of the participant. The study was kept open-label. One group received the tablet dapagliflozin 10 mg once daily orally before food. Its dose was gradually increased to 10 mg twice daily, then continued until the end of the study. The other group received a tablet of vildagliptin 50 mg orally once per day before food. Its dose was gradually increased to 50 mg twice daily orally, then continued until the end of the study. No crossover of the study drugs was allowed. Participants in both groups were treated with tablet metformin 500-2000 mg daily in single or divided doses before food. The endocrinologist did all the dose titrations.

At the baseline visit, we recorded sociodemographic parameters in a pre-prepared case record form. All participants underwent general physical and systemic examinations. Blood samples were collected to check hematological parameters and liver and kidney function tests. A thorough examination of the central nervous system was done to check for any neuropathy. The privacy of all participants was maintained, and precautions regarding the confidentiality of their clinical details were taken care of. The glycated hemoglobin (HbA1c), fasting blood glucose (FBG), and postprandial blood glucose (PPBG) values of each participant were noted at the baseline visit. After these proceedings, they received the drugs assigned to their groups free of cost. We asked for follow-up visits at 4, 12, and 24 weeks. The blood samples were collected at each follow-up visit to estimate FBG and PPBG. HbA1c was assessed after 12 and 24 weeks of initiation of study drugs. All assessments were done in the per-protocol (PP) population.

Based on the previous studies, the mean difference of HbA1c was considered for the calculation of the sample size of this study. Considering a mean difference of 1.5 in HbA1c from the baseline standard deviation of 0.3, 102 patients (51 in each group) were required to detect a change in HbA1c with a power of 80% at a two-sided significance level of 0.05. Considering 10% drop-out or loss to follow-up, a sample size of 112 patients (56 in each group) was finalized for this study. The categorical and continuous data were presented as number (percentage) and mean ± standard deviation, respectively. The intergroup and intragroup analyses were done using unpaired Student’s t-test and repeated measures ANOVA. A p-value < 0.05 was considered statistically significant. R software v. 4.1.1 (R Foundation, Indianapolis, IN) [12] was used for the data analysis and generation of the plots.

## Results

**Figure 1:**
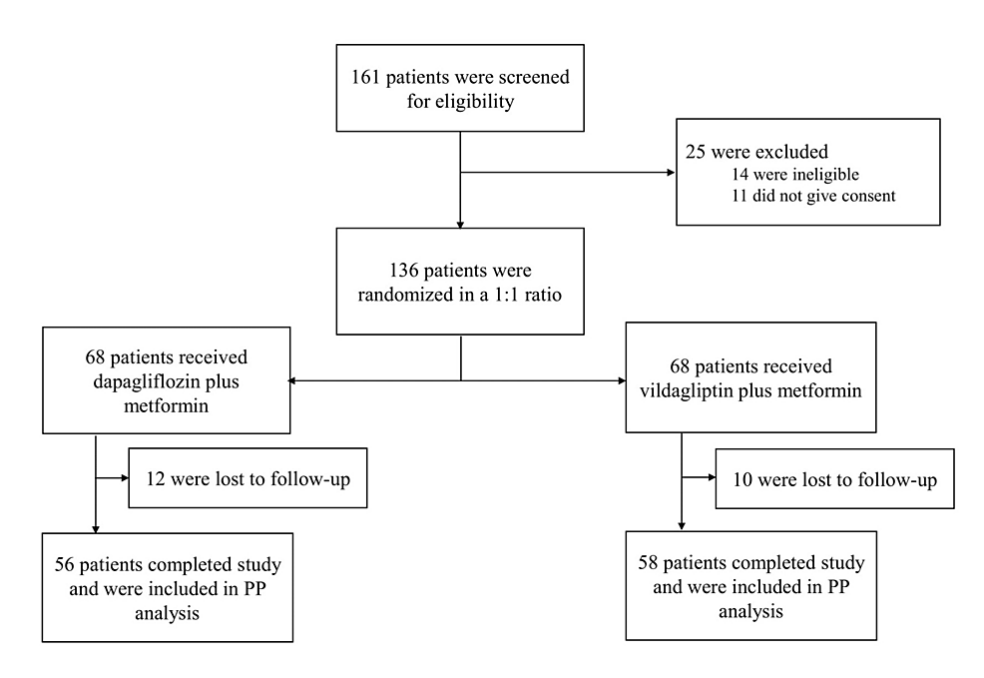
CONSORT diagram PP analysis: per-protocol analysis

For this study, 161 newly diagnosed type 2 diabetes mellitus patients were screened for eligibility in the Endocrinology OPD, KIMS, Bhubaneswar; 14 were found ineligible, and 11 withdrew consent before their enrolment. A total of 136 patients meeting the study criteria were enrolled. They were randomly assigned in a 1:1 ratio to either of the study groups. Out of them, 78 received dapagliflozin plus metformin, and 78 received vildagliptin plus metformin for 24 weeks. Twenty-two participants were lost to follow-up (i.e., 12 from the dapagliflozin group and 10 from the vildagliptin group). Finally, 114 participants completed the study and were included in the efficacy assessments as per the per-protocol (PP) analysis (Figure 1).

The baseline characteristics were similar in both groups (Table 1). The mean ages of the study participants of dapagliflozin and vildagliptin groups at the baseline visit were 40.83 ± 5.66 and 41.31 ± 5.34 years, respectively (p = 0.208). The mean HbA1c values of the study participants of the two study groups at the baseline visit were 8.22 ± 1.34 and 8.33 ± 1.56, respectively (p = 0.844). The mean FBG and PPBG at the baseline visit were 148.56 ± 16.34 and 151.71 ± 18.93 (p = 0.439), and 231.09 ± 28.12 and 228.47 ± 24.58 (p = 0.508), respectively.

**Table 1:** Baseline sociodemographic and clinical parameters of the study population The categorical and continuous data were expressed as n (%) and mean ± SD, respectively. HbA1c: glycated hemoglobin, FBG: fasting blood glucose, PPBG: postprandial blood glucose.

	Dapagliflozin plus metformin (n = 56)	Vildagliptin plus metformin (n = 58)	p-value
Age (years)			
Mean age	40.83 ± 5.66	41.31 ± 5.34	0.208
> 50 years	28 (50.00%)	28 (48.28%)	0.812
≤ 50 years	28 (50.00%)	30 (51.72%)	
Gender			
Male	29 (51.79%)	30 (51.72%)	0.647
Female	27 (48.21%)	28 (48.28%)	
Socioeconomic status			
Low	32 (57.14%)	36 (62.07%)	0.393
Middle	24 (42.86%)	22 (37.93%)	
HbA_1c_ (%)	8.22 ± 1.34	8.33 ± 1.56	0.844
FBG (mg/dl)	148.56 ± 16.34	151.71 ± 18.93	0.439
PPBG (mg/dl)	231.09 ± 28.12	228.47 ± 24.58	0.508
Serum creatinine (mg/dl)	0.73 ± 0.12	0.69 ± 0.17	0.713

**Figure 2:**
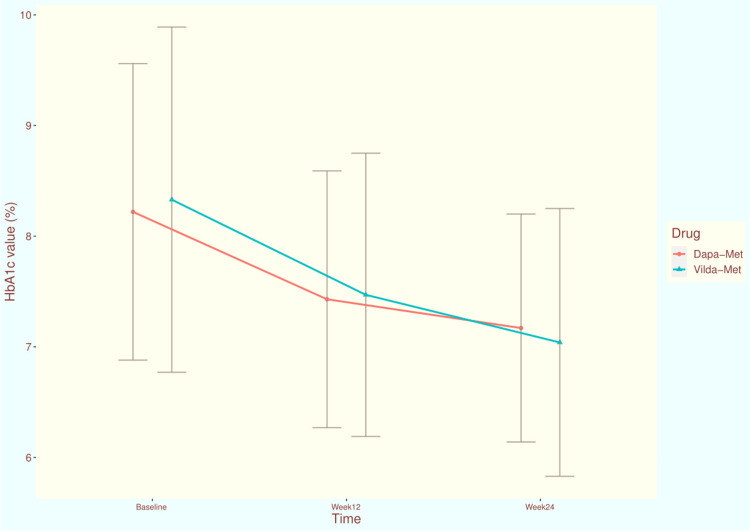
The HbA1c values of the study population at various time points of assessment The line diagrams with error bars represent the mean and standard deviation of HbA1c at various time points of assessments. HbA1c: glycated hemoglobin Dapa-Met: This study group's participants received dapagliflozin plus metformin for 24 weeks Vilda-Met: This study group's participants received vildagliptin plus metformin for 24 weeks.

The HbA1c values of the study population at each time point of assessment are illustrated in Figure 2. At the baseline visit, the mean HbA1c values of participants receiving dapagliflozin-metformin and vildagliptin-metformin were 8.22 ± 1.34 and 8.33 ± 1.56, respectively (p = 0.844). At 12 weeks, the scores got reduced to 7.43 ± 1.16 and 7.47 ± 1.28, respectively (p = 0.761). After 24 weeks of intervention, mean HbA1c values of dapagliflozin and vildagliptin groups were reduced to 7.17 ± 1.03 and 7.04 ± 1.21, respectively (p = 0.192). The mean change in HbA1c from baseline at 24 weeks were -1.19 (95% CI: -1.36 to -1.03) and -1.28 (95% CI: -1.37 to -1.18) in the dapagliflozin and vildagliptin groups, respectively (p = 0.213). The inter-group difference regarding the change of HbA1c values from baseline was clinically (not statistically) significant.

**Figure 3:**
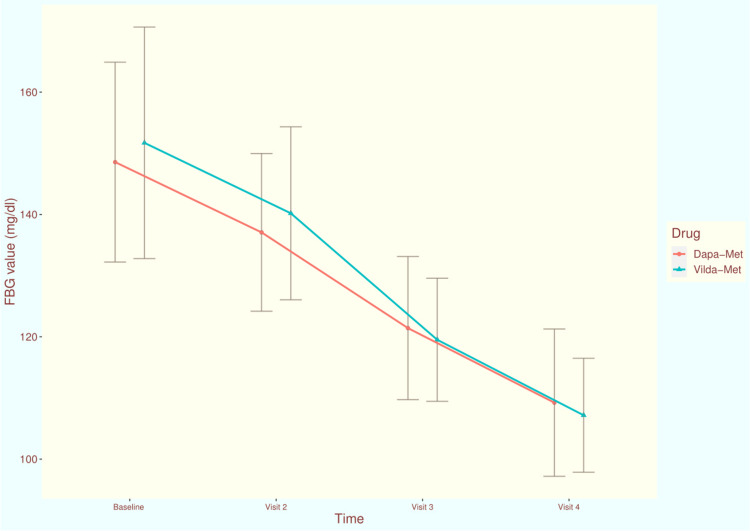
The FBG values of the study population at various time points of assessment The line diagrams with error bars represent the mean and standard deviation of FBG at various time point of assessments. FBG: fasting blood glucose, mg/dl: milligram per deciliter Dapa-Met: This study group's participants received dapagliflozin plus metformin for 24 weeks Vilda-Met: This study group's participants received vildagliptin plus metformin for 24 weeks.

The FBG values of the study population at each time of assessment are illustrated in Figure 3. At the baseline visit, the mean FBG values of participants receiving dapagliflozin-metformin and vildagliptin-metformin, were 148.56 ± 16.34 and 151.71 ± 18.93, respectively (p = 0.439). After four weeks of treatment, the scores were 137.07 ± 12.89 and 140.19 ± 14.14, respectively (p = 0.271). During the third visit at 12 weeks, the scores got reduced to 121.42 ± 11.70 and 119.51 ± 10.06, respectively (p = 0.326). After 24 weeks of intervention, mean FBG values of dapagliflozin and vildagliptin groups, were reduced to 109.23 ± 12.04 and 107.27 ± 9.32, respectively (p = 0.092). The mean change in FBG from baseline at 24 weeks were -38.76 (95% CI: -44.79 to -32.72) and -46.13 (95% CI: -51.24 to -41.02) in dapagliflozin-metformin and vildagliptin-metformin groups, respectively (p = 0.068). The inter-group difference regarding the change of FBG values from baseline was clinically (not statistically) significant.

**Figure 4:**
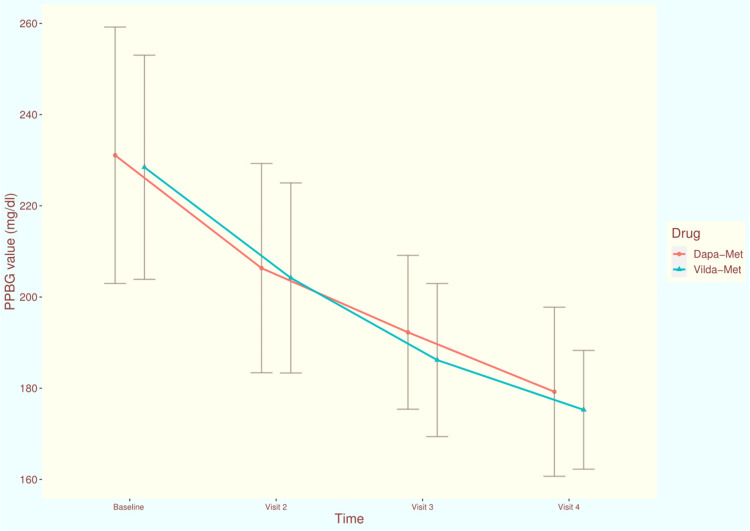
The PPBG values of the study population at various time points of assessment The line diagrams with error bars represent the mean and standard deviation of PPBG at various time points of assessments. PPBG: 2-hour post-prandial blood glucose, mg/dl: milligram per deciliter Dapa-Met: This study group's participants received dapagliflozin plus metformin for 24 weeks Vilda-Met: This study group's participants received vildagliptin plus metformin for 24 weeks.

The PPBG values of the study population at each time of assessment are illustrated in Figure 4. At the baseline visit, the mean PPBG values of participants receiving dapagliflozin-metformin and vildagliptin-metformin were 231.09 ± 28.12 and 228.47 ± 24.58, respectively (p = 0.508). After four weeks of treatment, the scores were 206.34 ± 22.95 and 204.19 ± 20.84, respectively (p = 0.724). During the third visit at 12 weeks, the scores got reduced to 192.27 ± 16.87 and 186.18 ± 16.78, respectively (p = 0.142). After 24 weeks of intervention, mean PPBG values of dapagliflozin and vildagliptin groups, were reduced to 179.23 ± 18.54 and 175.27 ± 13.02, respectively (p = 0.080). The mean change in PPBG from baseline at 24 weeks were -51.84 (95% CI: -62.89 to -40.78) and -53.56 (95% CI: -63.66 to -43.45) in dapagliflozin-metformin and vildagliptin-metformin groups, respectively (p = 0.137). The inter-group difference regarding the change of FBG values from baseline was clinically (not statistically) significant.

## Discussion

This prospective, randomized, open-label study focused on the efficacy of add-on dapagliflozin and vildagliptin to metformin after 24 weeks of intervention in patients with newly diagnosed type 2 diabetes mellitus. One group received the tablet dapagliflozin 10 mg once daily orally. Whenever required, its dose was gradually increased to 10 mg twice daily, then continued until the end of the study. The other group received a tablet of vildagliptin 50 mg orally once per day. In case of requirement, its dose was gradually increased to 50 mg twice daily orally, then continued until the end of the study. No crossover of the study drugs was allowed. Participants in both groups were treated with tablet metformin 500-2000 mg daily in single or divided doses. This study ascertained that vildagliptin add-on therapy was more efficacious than dapagliflozin adjuvant therapy in reducing glycated hemoglobin, fasting, and postprandial blood glucose. However, the intergroup differences in these contexts were not statistically significant.

After 24 weeks of intervention, mean HbA1c values of dapagliflozin and vildagliptin groups were reduced to 7.17 ± 1.03 and 7.04 ± 1.21, respectively (p = 0.192). The mean change in HbA1c from baseline were -1.19 (95% CI: -1.36 to -1.03) and -1.28 (95% CI: -1.37 to -1.18) in dapagliflozin and vildagliptin groups, respectively (p = 0.213). These findings were consistent with two network meta-analyses by Wang et al. [11] and Goring et al. [13]. On the contrary, a study by Del Prato et al. [14] suggested that a combination of SGLT2 inhibitor and metformin was more effective in long-term control of HbA1c than the combination of DPP-4 inhibitor and metformin. The mean change in FBG from baseline was -38.76 (95% CI: -44.79 to -32.72) and -46.13 (95% CI: -51.24 to -41.02), respectively (p = 0.068). Regarding PPBG values, mean change from baseline were -51.84 (95% CI: -62.89 to -40.78) and -53.56 (95% CI: -63.66 to -43.45) in dapagliflozin-metformin and vildagliptin-metformin groups, respectively (p = 0.137). Our observations regarding the change in FBG and PPBG values from baseline were concordant with that of recent studies by Son et al. [10] and Matthews et al. [15]. Furthermore, a retrospective real-world evidence study by Mohan et al. [16] suggested that a combination of vildagliptin and metformin in newly diagnosed diabetic patients attains a faster euglycemic state than other combinations.

Our study was strengthened by block randomization with stratification, frequent follow-up visits, and using an active drug as a comparator. However, there were a few limitations to our study as well: firstly, a smaller sample size, probably because of the multitudinous eligibility criteria of the study and the global Covid-19 pandemic; secondly, an open-label study design could have created room for recall bias, mainly during the safety assessments. Thirdly, we could not gather extensive data regarding co-morbidities and other concomitant medications. We did not assess the effects of those drugs.

## Conclusions

We conclude that vildagliptin was more efficacious as an adjuvant to metformin in the treatment of type 2 diabetes mellitus as compared to dapagliflozin. We suggest further studies with a more heterogeneous study population in order to understand the long-term effects of these drugs.
